# Effect of Zinc Supplementation in Children with Acute Diarrhea: Randomized Double Blind Controlled Trial

**DOI:** 10.4021/gr2009.06.1298

**Published:** 2009-05-20

**Authors:** Sangita S Trivedi, Rajesh K Chudasama, Nehal Patel

**Affiliations:** a Pediatric Department, Government Medical College, Surat, India; b Community Medicine Department, Government Medical College, Surat, India

**Keywords:** Acute diarrhea, Zinc, Placebo, Frequency of stool, Randomized controlled trial, Blinding

## Abstract

**Background:**

To test the hypothesis that daily supplementation of zinc has any effect on clinical course of acute diarrhea, i.e. frequency of stool, on stool amount and duration of acute diarrhea.

**Methods:**

In a randomized double blind placebo controlled trial, 117 children aged 6 months to 59 months in a medical college hospital, with acute diarrhea of less than 14 days were assigned by permuted block design 1:1 to receive intervention of zinc supplemented syrup (n = 60) or placebo syrup (n = 57).

**Results:**

Baseline characteristics were similar in both the groups. Mean age in zinc supplemented group was 22.14 ± 16.68 months and in placebo group 25.66 ± 17.02 months. Reduction in stool frequency per day was found 62% in zinc supplemented group and 26% reduction was found in placebo supplemented group with obvious difference of 36% between these two groups from day 1 to day 3 and day 5, which was found statistically highly significant. Similarly, significant difference was observed for reduction in amount of stool per day from day 1 to day 3 and day 5 with obvious difference of 45% between the study groups.

**Conclusions:**

Oral zinc administration in acute diarrhea reduces the frequency of diarrhea and output of stool by changing the natural course of acute diarrheal disease, causes early normalization of stool consistency, early recovery and decreases total duration of hospital stay. Zinc supplementation is simple, acceptable and affordable strategy which should be considered in management of acute diarrhea.

## Introduction

Diarrheal disease constitutes a leading cause of morbidity and mortality among children less than five years of age in developing countries [1]. In recent years, the major advance in the treatment of acute gastroenteritis in children was the introduction of oral rehydration solution (ORS) in the early stages of illness [2]. Significant proportions of children who suffer from diarrhea are malnourished with depleted micronutrient stores. Diarrhea also leads to excess loss of micronutrients such as zinc and copper. Therefore children with marginal nutritional status are also at the greatest risk of developing zinc and copper depletion with an episode of diarrhea [3]. Zinc has direct effect on intestinal villus, brush border disaccharidase activity and intestinal transport of water and electrolytes [4]. Zinc supplements given during diarrhea reduce the duration and severity of treated episodes [5] and, if given for total 14 days during and after diarrhea, can reduce the incidence of diarrhea in subsequent two or three months [6]. Therefore, we conducted a randomized, double blind, placebo controlled trial to test the hypothesis that daily supplementation of zinc has effect on frequency and amount of stool and shortens the duration of acute diarrhea in children of 6 months to 5 years of age while treating acute diarrhea.

## Subjects and Methods

### Study area and population enrollment

The study was conducted at pediatric department (single centered), Government Medical College and Hospital, Surat, Gujarat, India as per the Helsinki Declaration and after approval by the human research ethical committee of the Government Medical College, Surat. In present trial, all children aged 6 months to 59 months who presented and admitted to hospital (only inpatients) with more than three unformed stools in 24 hours and diarrheal duration of < 14 days were eligible after written informed consent. All patients without any signs of dehydration were also monitored in diarrheal treatment unit of the hospital. A child could be enrolled only once. Child with any other significant systemic illnesses and severe malnutrition was excluded. Any child receiving systemic or oral antibiotics, multivitamins, iron, antimotility drugs, pre- and probiotics and other drugs before or after admission were also excluded.

### Baseline assessment

The following baseline data was collected, including diarrheal duration, frequency of stool, amount of stool, character of stool (watery, mucoid or bloody), age, sex, maternal education, family income, diet of child (mostly breastfed, partially breastfed, other milk, mixed feeding or family diet), immunization status, history of fever or vomiting, prior use of ORS, prior use of medications and nutritional status. All patients with dehydration were provided oral rehydration solution as per WHO guidelines. Height and weight were measured by using UNICEF Detecto Scale by a trained person. Infantometer was used to measure length of children below 24 months of age. Stool output was monitored as soon as patient was admitted, and weighed as and when required by using electronic weighing machine during hospitalization by trained personnel. All the information was recorded on predesigned and pretested proforma.

### Interventions

The liquid preparation containing zinc or a placebo used as syrup in present trial contained (1) water and sugar at 50% concentration, (2) mix fruit flavored agent, and (3) Na Benzoate (0.2%) as a preservative. The zinc syrup contains same vehicle with zinc sulfate with concentration of 2 mg elemental zinc per milliliter (ml). Both the syrup bottles containing either zinc or a placebo were identical in appearance and taste. Syrup bottles were given to each child’s mother and kept with them. Patients were given double the recommended daily allowance of zinc, as 5 ml (10 mg) for child less than one year and 10 ml (20 mg) for child more than one year in two divided doses per day after hospital admission, and continued till they were get cured and discharged.

### Objectives

Present trial was done to test the hypothesis that daily supplementation of zinc have any effect on clinical course of acute diarrhea, frequency of stool, on stool amount and duration of acute diarrhea in children age group 6 months to 5 years of age.

### Outcome variables

The primary outcome was frequency of diarrhea per day from the time of onset. A diarrhea was considered as a 24 hour period with passage of at least three unformed stools and this episode was considered terminated on the last day of diarrhea followed by 24 hour diarrhea free period. The amount of stool was measured as primary outcome variable from day of admission to day 5 or to the day of discharge. The number and proportion of patients with diarrhea less than 14 days and mean length of hospital stay was also estimated. Serum protein, albumin, alkaline phosphatase values at the time of admission, impact of zinc supplementation on stool amount during acute diarrhea were also estimated by using biochemical investigation.

### Sample size and follow up

A prospective study was conducted for one year, started from August 1, 2007 to July 31, 2008. The purpose of selecting one year as study period was to cover all the seasons throughout the year and avoid any selection bias and seasonal influence. All patients having acute diarrhea in the age group of 6 months to 6 years admitted in hospital during this period were enrolled in the study. Total 1492 pediatric patients were admitted in wards of pediatric department during this time period at Government Medical College, Surat. Out of these, 196 admission were belongs to 6 months to 5 years of age group with diarrhea. Total 117 patients were enrolled in this study out of 196 admissions of study group throughout the year. Remaining 79 patients in same age group were excluded because they were having concomitant other illness, received some systematic or oral antibiotic before admission or any kind of other drugs, received preadmission multivitamins/iron, antimotility drugs, pre and probiotics like lactobacilli, etc. Parents of all 117 patients have given written consent and were enrolled in this study without refusal to participate, of which 60 patients were allocated to experimental group and 57 patients to placebo group after randomization. They were followed up in hospital after admission on day 1, 3, and 5. At the end of follow up after 5 days in the hospitalized patients, 44 patients were remained in experimental group and 44 patients in placebo group and included in analysis.

### Randomization – sequence generation, allocation concealment & implementation

Children who fulfilled the inclusion criteria were enrolled into two treatment groups, experimental and placebo group. The sequence to enroll children in either group was generated by using restricted randomization by using permuted block design of 1:1 to ensure that the comparison groups will be of approximately equal size. Stratification was done as per the age, and socioeconomic status of children. Age was grouped in 6 - 12 months, 13 - 36 months and 37 - 60 months groups. Modified Prasad’s socioeconomic classification [7] was used to determine socioeconomic status of children. Patients were allocated a specific numbered bottle of either zinc or placebo syrup without revealing its identity. The person who made the sequence generation was not monitoring the study groups. The sequence code numbers were kept in a sealed envelop with one of the senior officer who identified the groups after completion of the study. After recovery from illness, children were discharged and parents were advised to continue the given syrup for total 14 days along with home available routine diet. Due care was taken to provide adequate amount of appropriate bottle of zinc/placebo syrup at discharge of patients.

### Blinding

Double blinding technique was used in present study. All study participants and personnel including care providers, evaluators and monitors were blinded to treatment assignment for the whole duration of the study to avoid any type of bias. Patient blinding was evaluated by asking questions to patients and their parents to indicate which type of treatment they believed they had received like special medicine containing syrup or a routine syrup during follow up on day 3, day 5 or at time of discharge if earlier. But no patient replied that they have any idea about the type of syrup they are receiving. Similarly, blinding study personnel including care providers were asked questions that which type of syrup they believed that they are giving to the patients. When they answered either zinc or placebo syrup, they were asked to indicate how they have identified, but no one has correctly identified.

### Statistical analysis

All data analysis was done as per the preestablished analysis plan. Exploratory data analysis was carried out to assess the distribution of study variables. Baseline characteristics of treatment groups were entered in computers, compared and analyzed by using Epi Info 6 software. Mean and standard deviations (SD) were calculated and statistical significance was tested by using Student’s t test. Two sided significance tests were used throughout.

## Results

### Flow and follow up of participants

**Figure 1:**
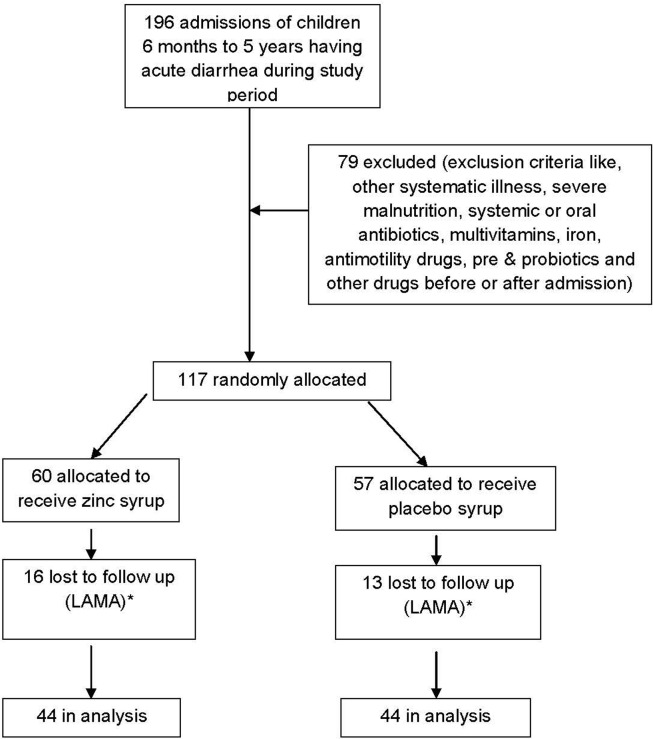
Flow diagram of a trial of zinc supplementation in children with acute diarrhea. * LAMA- Leave against medical advice

Total 1492 patients were admitted to pediatric department at Government Medical College, Surat during study period. Out of these, 196 patients belonged to study group (Fig. 1). Due to exclusion criteria, 79 children were excluded from the trial. So, 117 children were allocated for the trial. Among these, 60 were allocated in experimental group receiving zinc syrup and 57 were allocated in placebo group receiving placebo syrup. During hospital follow up, 16 patients were lost to follow up in experimental group and 13 in placebo group as they left hospital against medical advice. Finally, 44 patients were remained for analysis in both the groups. Mean age in zinc supplemented group was 22.14 ± 16.68 months and in placebo group 25.66 ± 17.02 months.

### Analysis

Baseline characteristics of study group children are shown in Table 1. Majority participants belong to age 6 months to 36 months. There was an almost equal socioeconomic distribution among study participants. Diarrhea frequency more than 10 times per day with less then 7 days duration on admission was found mainly in both study groups.

**Table 1:** Baseline characteristics of zinc supplemented and placebo group children

Variables	Zinc supplemented group (n = 44)		Placebo group (n = 44)	
	No.	%	No.	%
Age in months				
6 - 12	19	43.2	17	38.6
13 – 36	18	40.9	18	40.9
37 – 60	7	15.9	9	20.5
Sex of child				
Male	23	52.3	16	36.4
Female	21	47.7	28	63.6
Socioeconomic status				
Upper	12	27.3	20	45.5
Middle	18	40.9	14	31.8
Lower	14	31.8	10	22.7
Diarrhea frequency/day on admission				
≤ 5	4	9.1	2	4.5
6 - 10	8	18.2	13	29.6
> 10	32	72.7	29	65.9
No. of days for diarrhea on admission				
≤ 7	41	93.2	41	93.2
8 – 14	3	6.8	3	6.8
Diarrhea with fever				
Yes	18	40.9	15	34.1
No	26	59.1	29	65.9
Diarrhea with vomiting				
Yes	36	81.8	36	81.8
No	8	18.2	8	18.2

Table 2 shows impact of zinc supplementation on frequency of stool per day during follow up on day 1, day 3 and day 5 during acute diarrhea. Results showed that in all the age groups, stool frequency per day significantly reduced from day 1 to day 3 and day 5 in zinc supplemented group compare to placebo group, which was primary outcome variable. Reduction in stool frequency per day was found 62% in zinc syrup supplemented group and 26% reduction was found in placebo syrup supplemented group with obvious difference of 36% between these two groups (p < 0.01).

**Table 2:** Impact of zinc supplementation on frequency of stool per day during follow up as per mean ± SD

Age group	Zinc supplementation group (n = 44)			Placebo group (n = 44)		
	Day 1	Day 3	Day 5	Day 1	Day 3	Day 5
6-12	9.05 ± 4.89	3.75 ± 2.59*	2.90 ± 1.92*	11.19 ± 7.21	8.57 ± 7.78	3.4 ± 3.53*
13-36	11.78 ± 7.25	3.89 ± 2.91*	1.92 ± 1.32*	11.89 ± 9.64	8.37 ± 6.39	3.54 ± 3.47*
37-60	8.28 ± 7.25	5.4 ± 3.43	1.91 ± 1.21*	7.77 ± 2.10	6.5 ± 1.92	3.59 ± 3.43
Total	10.7 ± 6.38	4.06 ± 2.82*	2.32 ± 1.58*	10.86 ± 7.71	8.05 ± 6.26	3.40 ± 3.34*

* Indicates p < 0.01, percent reduction in frequency of stool/day- Zinc: 62%, Placebo: 26%, difference: 36%.

Effect of zinc supplementation on stool amount per day is shown in Table 3. All the stool evacuations were collected and weighed by trained personnel in hospital. Significant reduction in per day stool amount as primary outcome was observed from day 1 to day 3 and day 5 while comparing zinc supplemented group and placebo group. Serum protein, albumin, and alkaline phosphatase, and zinc were also checked in all the age groups by doing biochemical investigations. Serum alkaline phosphatase was found low in study groups. Serum zinc level was found low at time of admission but on third day when tested again, shown rise in serum level in zinc supplemented group. Body weight and mid arm circumference measurement at time of admission and at time of discharge was done but was statistically not significant. Compliance of every enrolled patient was found adequate throughout the study. All patients were observed for any kind of adverse effects during trial by monitoring each patient by examining and by asking patients for any adverse effect development but no adverse effects were reported in any of the study participants throughout the trial.

**Table 3:** Impact of zinc supplementation on amount of stool/day during management of acute diarrhea as per mean ± SD

Age group	Zinc supplementation group (n = 44)			Placebo group (n = 44)		
	Day 1	Day 3	Day 5	Day 1	Day 3	Day 5
6-12	161.8 ± 195.5	116.4 ± 18.8	43.8 ± 31.9*	164.7 ± 182.4	226.7 ± 419.5	158.0 ± 239.0
13-36	345.5 ± 296.2	181.1 ± 220	103.3 ± 78.1*	232.7 ± 273.2	195.1 ± 187.4	139.7 ± 147.9
37-60	261.0 ± 232.0	77 ± 54.3	113.3 ± 118.4	68.7 ± 46.1	38.6 ± 51.8	131.2 ± 163.5
Total	259.6 ± 272.2	144.4 ± 175*	80.7 ± 72.8*	173.4 ± 215.9	183.9 ± 292.9	148.8 ± 192.3

*Indicates p <0.05, percent reduction in stool output/day, Zinc: 45%, Placebo: 0%, difference: 45%.

## Discussion

Present study examined several aspects of zinc supplementation. Our study data indicates that supplementing the children with acute watery diarrhea in age group of 6 - 60 months with double the recommended daily intake of zinc during the period of acute illness has significantly decreased the frequency of diarrhea and amount of stool output, and improved the early normalization of stool consistency on third day of zinc supplementation. Considering the recovery parameters, it is acknowledged that stool output has decreased significantly in total patients supplemented with zinc (p < 0.05) on day 3 and day 5 as compared to control placebo group. Present study also showed significant reduction in stool frequency (36%) and in stool output (45%) by zinc supplementation on third day. The findings of present study indicate that zinc supplementation in acute diarrhea effectively reduces both frequency of diarrhea and output of stool. Baqui AH et al [8] reported in their study of zinc and copper supplementation on Bangladeshi children, that those who received zinc supplementation during and after diarrhea had 24% shorter duration of diarrhea, 15% lower incidence of diarrhea, and a trend suggesting fewer diarrhea related hospital admissions.

Moreover, the diarrheal duration and frequency, primary concerns of the mother also do provide some valuable information regarding recovery. As is seen that the diarrheal frequency and duration was comparable in both groups initially, but on third day the zinc supplemented group has shown faster recovery in frequency and duration of diarrhea. In contrast, Patel AB et al [3] have reported that most important predictor for duration of diarrhea in children was the severity of the disease at enrollment, and not the supplementation, but in contrast present study showed no such role of disease severity. The reduction in duration of diarrhea episode is consistent with earlier studies [9, 10]. A meta-analysis of five studies of zinc treatment for acute diarrhea found a summary estimate for reduction in duration of 16% [5]. Possible mechanisms for the effect of zinc treatment on the duration of diarrhea include improved absorption of water and electrolytes by intestine, faster generation of gut epithelium, enhanced immune response, leading to early clearance of diarrheal pathogens from intestine [11, 12]. Rahman MM et al [13] have reported that combined zinc and vitamin A synergistically reduced the prevalence of persistent diarrhea and dysentery.

Serum alkaline phosphatase measurement has been suggested as an important surrogate marker of zinc status in human [14]. In present study, it has been shown that serum alkaline phosphatase was low in all patients, may be correlating with zinc deficiency, but we were not able to do the follow up study on the seventh and fourteenth day supplementation. This is the limitation of our study that rise in serum alkaline phosphatase with serial zinc supplementation was not followed as supported by other studies [15]. But as we have measured serum zinc on third day of supplementation with zinc syrup, it was seen that serum zinc has shown significant rise on third day, that suggests that sufficient absorption and replenishment of body zinc occurred with the double dose of zinc used. Both the groups were not given any other multivitamin or micronutrient supplement which may interfere with absorption of others and study results by their effect.

Present study was hospital based and only admitted patients to the pediatric wards were enrolled in the study which leads to selection bias, excluding patients treated on outpatient and that was the limitation of present study.

In conclusion, oral zinc administration in acute diarrhea decreases the frequency of diarrhea and output of stool by changing the natural course of acute diarrheal disease, causes early normalization of stool consistency, early recovery and decreases total duration of hospital stay. Zinc supplementation is simple, acceptable and affordable strategy which should be considered in management of acute diarrhea.
